# Numerical investigation of compressible flow around nose cone with Multi-row disk and multi coolant jets

**DOI:** 10.1038/s41598-023-28127-9

**Published:** 2023-01-16

**Authors:** Reza Iranmanesh, As’ad Alizadeh, M. Faraji, Gautam Choubey

**Affiliations:** 1 Faculty of Civil Engineering, K.N. Toosi University of Technology, Tehran, 158754416 Iran; 2 Department of Civil Engineering, College of Engineering, Cihan University-Erbil, Erbil, Iraq; 3 Department of Mechanical Engineering, Babol Noshirvani University of Technology, Babol, Iran; 4 Department of Mechanical & Aerospace Engineering, Institute of Infrastructure Technology Research and Management (IITRAM), Ahmedabad, Gujarat 380026 India

**Keywords:** Aerospace engineering, Mechanical engineering

## Abstract

Due to sever aerodynamic heating, the protection of forebody of scramjet is crucial for hypersonic flight. In present work, a new cooling system is proposed and investigated for the protection of nose cone at hypersonic flight. Computational fluid dynamic is used for the simulation of the lateral and axial coolant jet released from the spike at high-velocity condition. The primary goal is to find optimum jet location for efficient cooling of nose and spike assembly. Influence of two coolant jets (Carbon dioxide and Helium) on the mechanism of cooling system are fully investigated. For simulation, RANS equations are coupled with species transport equation and SST turbulence model. Two different jet configurations (axial disk positions) are investigated to obtain efficient condition for protection of nose cone at hypersonic flight. Our results indicate that the presence of the spike on the nose cone decreases pressure up to 33% on the main body and the shifts the maximum pressure to higher angles because of the deflection of the air stream. Maximum pressure drops about 50% by injection of the coolant disk jet (C2) at angle of 55 deg.

## Introduction

The cooling system for the thermal control of aerodynamic heating is the main issue for the design of forebody of high-speed vehicles and shuttles. Considerable efforts have been done to find efficient solution for this issue and some practical and theoretical techniques have been proposed and investigated in this regards^1,2^. The complex feature of the hypersonic flow nearby the nose cone is important challenge for the evaluation of the thermal efficiency of these proposed techniques^3,4^. Besides, the production of the shock with air dissociation also intensifies the complexity of the flow physic in the vicinity of the nose cone^5^.

Since the main concept of the forebody is to reduce drag reduction, a new methodology should consider this for thermal management of aerodynamic heating. In fact, the reduction of the both heat and drag should be balanced for the efficient model^6,7^. The mechanical device of spike is the most conventional practical model for the thermal reduction of the nose cone at hypersonic flow. In this technique, the flow separation occurs in the tip of spike and high temperature region is produced near the spike^8,9^. Since the spike reduces the drag force and heating on the nose cone, it is popular and practical in the real applications. The formation of shock and value of heat transfer is proportional with the shape and length of spike^10^. The strength and interaction of the bow shock produced in front of the spike is important for the thermal load on the main body. Previous works has extensively investigated various characteristic of mechanical spike to achieve optimum geometry of this technique^11–13^.

The application of the multi-row disk on the spike also enhances thermal performance of the spike^14,15^. In this method, the recirculation flow is produced in the gaps and this improves the heat transfer in the vicinity of the spike. Besides, the deflection of the blow shock is managed via size of disk and this could avoid the deflection of the bow shock on the main body^16,17^.

The injection of the coolant from the nose cone is also known as second technique for the thermal protection of the forebody of hypersonic vehicles^18,19^. In this concept, transient jet is released and this push the bow shock into the upstream. Besides, the low temperature of the coolant reduces the temperature of gas nearby the cone and consequently, heat transfer into the main body decreases^20,21^. Besides, the thermal conductivity (Cp) of the flow is changes by the injection of secondary gas and this is also effective on protection of the nose cone. Although this approach is not practical yet, it offers significant data about the mechanism of heat production via the aerodynamic heating process^22–24^.

**Figure 1:**
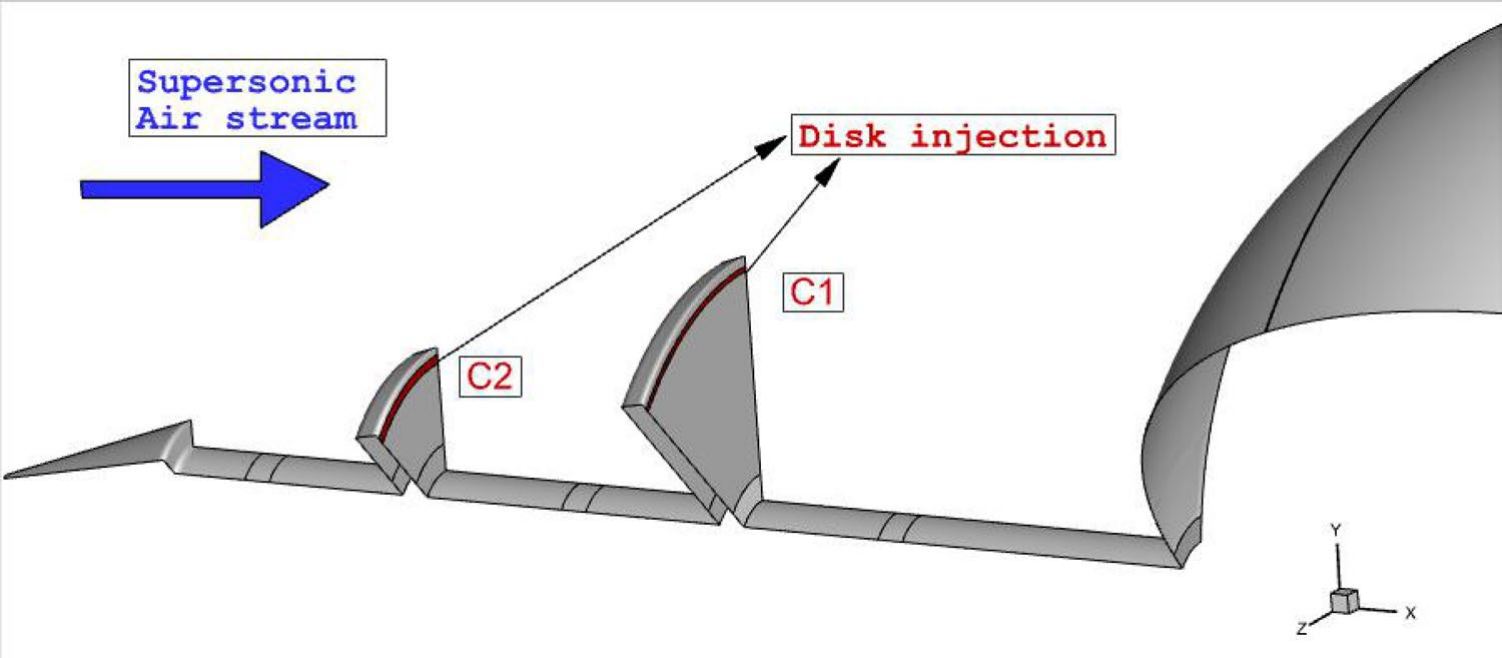
Selected model with proposed injection system.

Recently, the hybrid technique becomes popular for the thermal protection of the nose cone at hypersonic flow^25,26^. Combination of fluidic (opposing jet) and mechanical (spike) could help aerospace engineer to achieve higher performance in thermal and drag reduction^27–29^. The injection of the coolant jet from nose cone with multi-row disk is proposed in present work as displayed in Fig. 1. In the suggested technique, the cooling of the nose is done via injection of coolant from the spike while drag reduction is attended by the spike. This innovative method tries to apply both advantageous for the reduction of drag and heat on the nose and spike.

In this article, comprehensive three-dimensional studies have been done for the analysis of hybrid fluidic and mechanical devices for the thermal protection of the main body at hypersonic flight. Effects of the jet location and coolant types on the thermal load of spiked nose cone with the multi-row disk are fully investigated. CFD approach is used for the visualization of the compressible flow near the proposed configuration. Flow feature around the nose and heat flux rate on the main body and spike are investigated.

## Governing equations and computational technique

The simulation of the compressible flow around the nose cone is almost done via solving RANS equations^30–32^. For the simulation of helium and Carbon dioxide injection as secondary species, species transport equation is also couple as energy equation since latter is essential for the modeling of the shock inside the domain^33,34^. Due to high velocity of the air flow, the second order upwind scheme is used for the discretization of the convection terms of governing equation. Flow gas is assumed ideal gas and reactions and dissociations are not considered in this work^35,36^. Due to high speed flow situation, SST turbulence model is used for the calculation of viscosity in our study. For calculation of the heat capacity, the mixing law is applied^37–39^.

In this work, inflow is pressure farfield with M = 5.0, Pinf-2550 and Tinf = 221 K. Helium and carbon dioxide are chosen for as coolant jets with sonic condition at Ts = 300 K. Pressure outlet is extrapolated from the results of inside domain. The spike and main body is assumed wall with constant temperature of 300 K. The length of spike is equal to diameter of the main body. Two positions on tip of disk and three locations on the stem of spike are chosen for injection of the coolant jet. The area of these injector is equal to have identical mass flow rate for comparison of these configurations. To reduce the computational cost, only 45 degree of three full model is selected as domain for the simulations. Hence, symmetry condition is applied for two sides of domain^40,41^.

**Figure 2:**
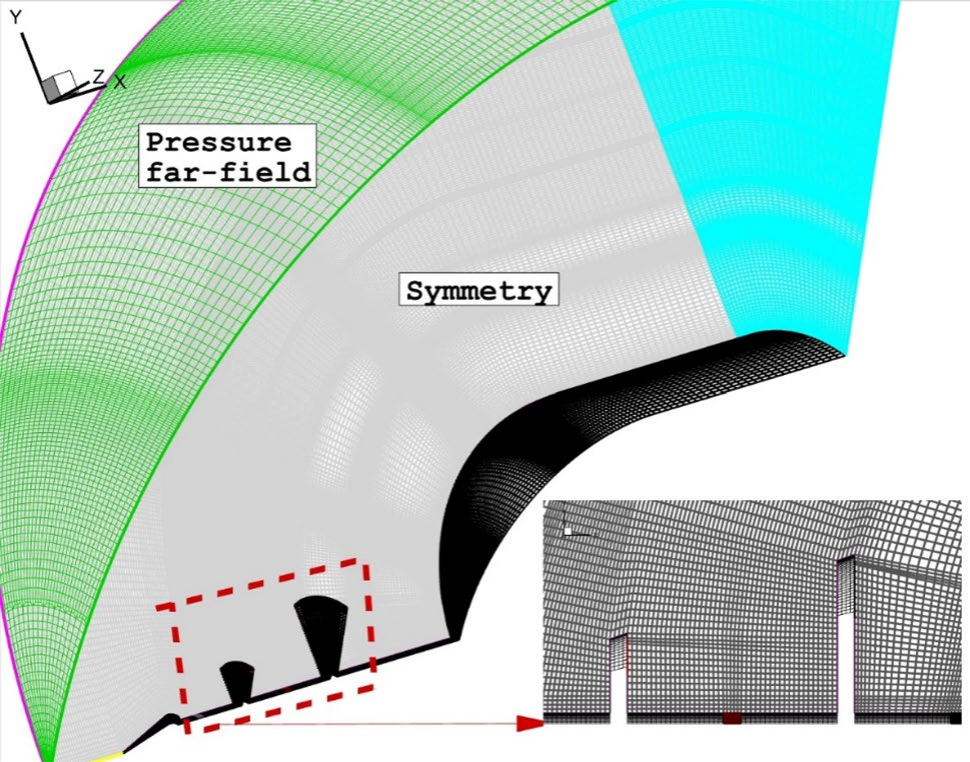
Grid production.

The grid production is done with specific considerations nearby the injectors and tip of spike and disk where main shock interaction and sever heating occur^42,43^. As displayed in Fig. 2, structured grid is used and this is mainly because of strong shock interactions and high temperature regions in our domain. Besides, the grid distribution shock be uniform to avoid error diffusion in our simulations. For grid independency analysis, four grid resolutions are generated and simulated in the first step. Comparison of the heat load on the main body are done for produced grids (Table 1) and it is found that fine grid with 1,628,000 cells. 74 h are computational time and residual for convergence is 10e − 4.

**Table 1:** Grid details.

Model	Grid number	Average Stanton numb. on blunt cone (θ = 30)	Average Stanton on numb. blunt cone (θ = 60)
Coarse grid	680,000	0.00212	0.00618
Normal grid	960,000	0.00245	0.00637
Fine grid	1,320,000	0.00251	0.00651
Very fine grid	1,680,000	0.00253	0.00653

## Results and discussion

**Figure 3:**
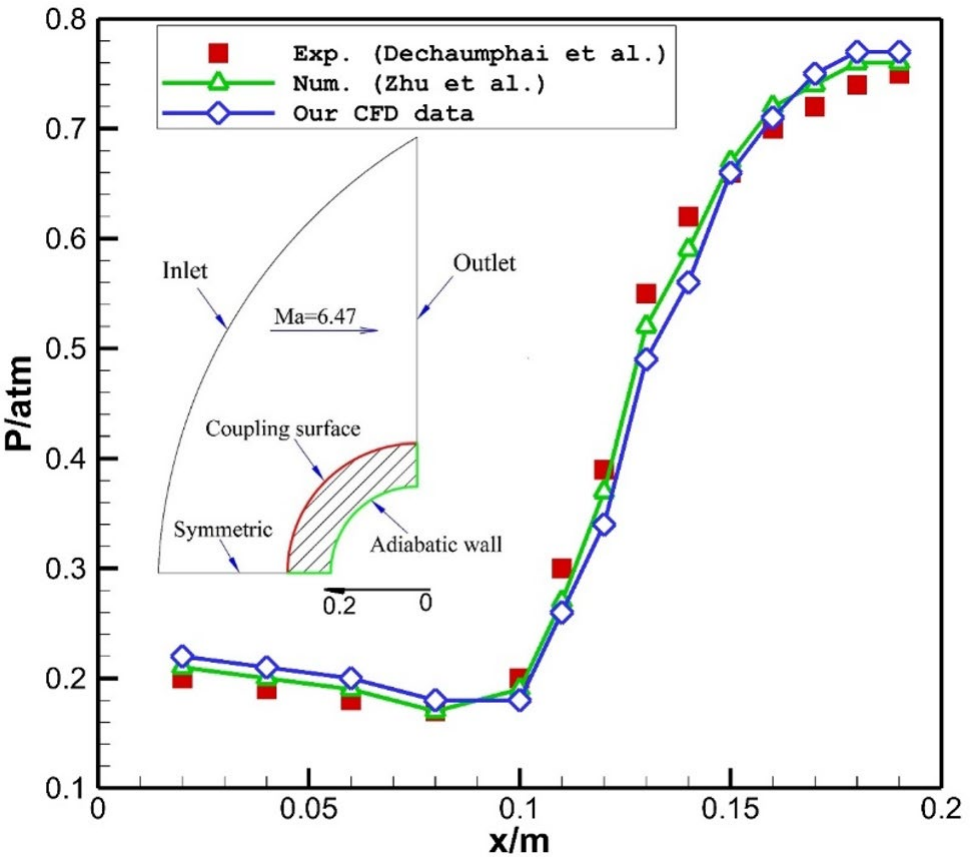
Validations^55^.

The comparison of our computational results with experimental work of Dechaumphai et al.^44^ are presented in Fig. 3. In this plots, changes of the normalized pressure on the main body of nose cone without spike are done. Besides, results of numerical study of Zhu et al.^39^ are also presented in this figure. It is observed that the average deviation of our data with other works is less than 6%. Validation of computational studies have been done in several previous works^45–55^

**Figure 4:**
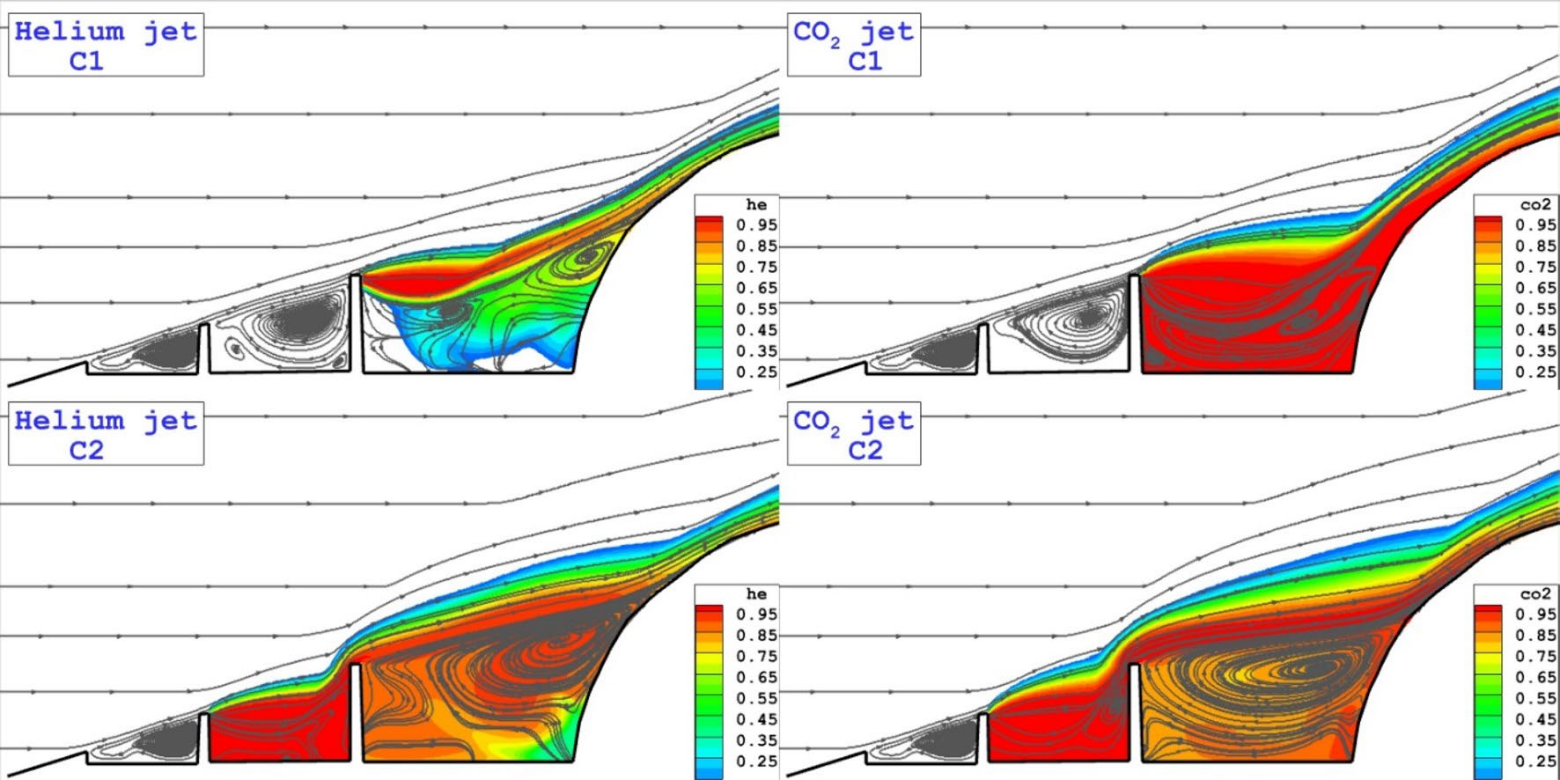
Comparison of the coolant mass fraction and flow stream for the injection system from the tip of disks.

Figure 4 displays the flow stream and concentration of the coolant gas (He and CO_2_) for injection system released from the tip of disk located on the spike. As expected, the formation of the circulations in the cavity is main flow physic of the proposed configurations. Comparison of these two coolant gases for Cl model indicates that the helium jet tends to moves with main stream while CO_2_ jets remains in the cavity of domain. In C2 model, the flow stream and concentrations of the coolant are almost identical since the circulation strength after the first disk is high enough.

**Figure 5:**
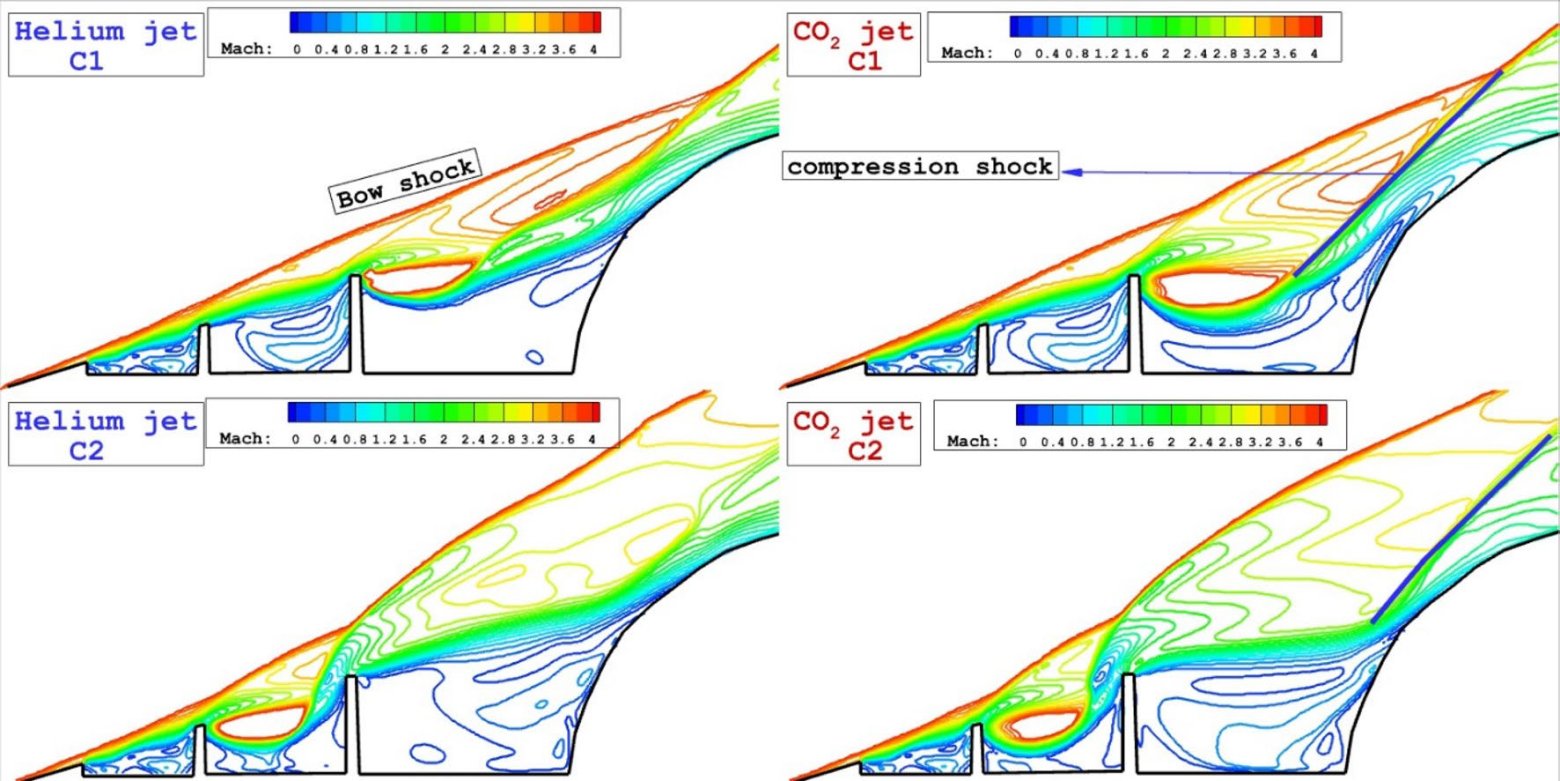
Comparison of Mach contour for the injection system from the tip of disks.

The main feature of the shock interactions of these two models are displayed in Fig. 5. In Cl model, the angle of comparison shock of CO_2_ jet is higher than that of Helium jet and this shows that the interactions of CO_2_ jet with main body is high in this configurations. In C_2_ model, helium jet deflects the main stream and limited interaction between shear layer and main body is noticed. However, the angle of bow shock is less in CO2 jet and deflection occurs on the shoulder of nose cone.

**Figure 6:**
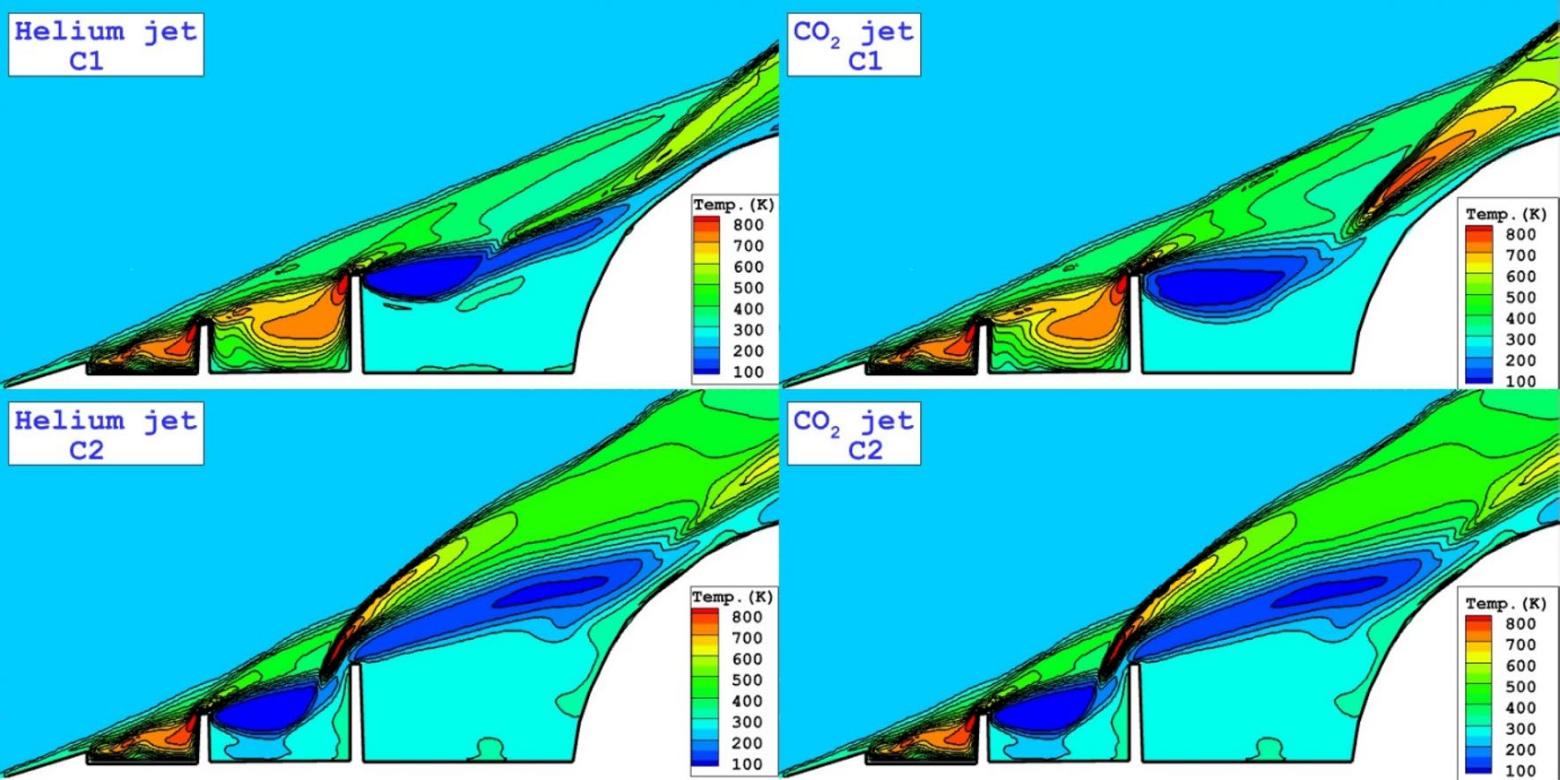
Comparison of temperature for the injection system from the tip of disks.

To notice the interactions and strength of the shock, temperature contours are these two tip injection system are displayed in Fig. 6. In the region with high temperature, the shock deflection is observed. Besides, the production of the high temperature region near the main body indicates the compression shock which increases the heat transfer into the main body. Helium jets deflect the main supersonic more than CO_2_ jets and this avoid formation of the high temperature area in the vicinity of the main body.

**Figure 7:**
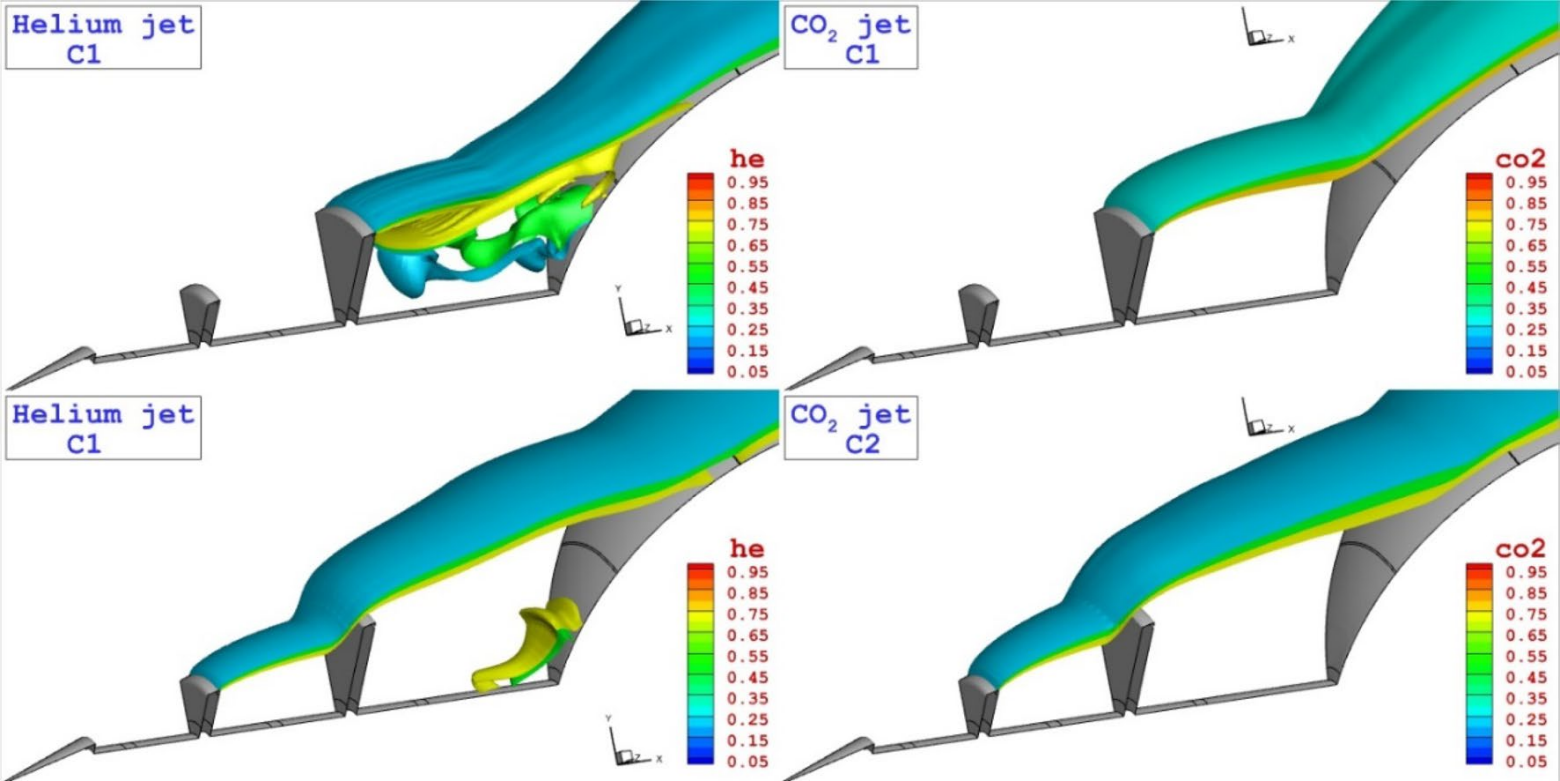
Three-dimensional flow feature with coolant distribution.

Three-dimensional flow structure of these two injection system are displayed in Fig. 7. The formation of the coolant layer represents the mechanism of the gas dispersion in the circulation regions and cooling mechanism in these configurations. Due to strong barrel shock of CO_2_ jet, the coolant layer has less deflection in cavity.

**Figure 8:**
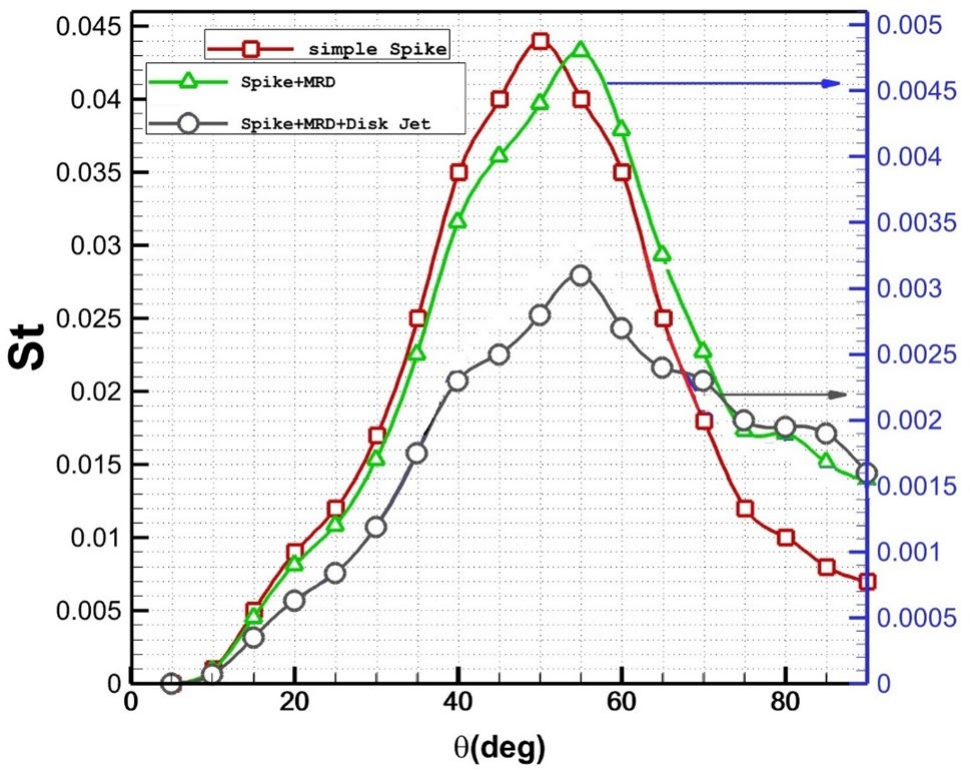
Heat transfer on the main body and disk in different conditions.

The Influence of the disk injection (C1) on the thermal distribution on nose cone are plotted in Fig. 8. It is noticed that the application of the multi-disk substantially decreases the Stanton number on the main body. In addition, the usage of the disk jet also decreases 37% maximum Stanton number on the main body.

**Figure 9:**
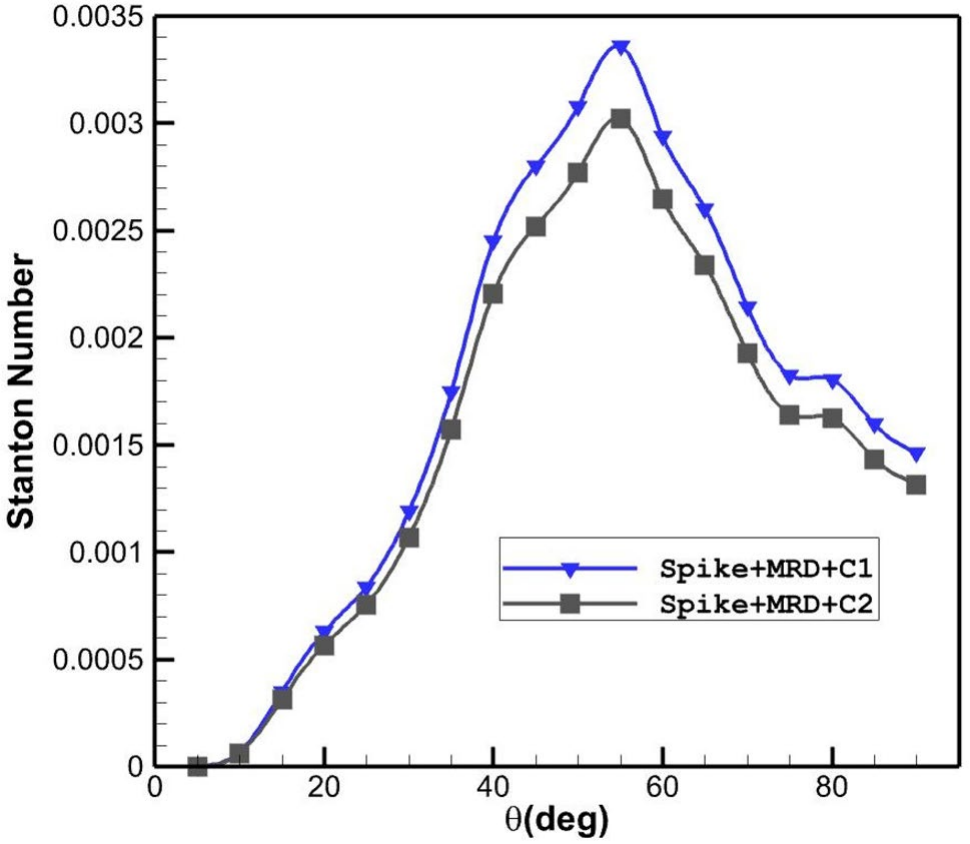
Distribution of Stanton number along main body of the different disk helium injection systems.

Effects of helium jet locations (disk 1 and 2) on Stanton distribution of the main are displayed in Fig. 9. The change of the Stanton number indicates that the heat transfer on the main body improves when injection of the coolant is near the main body as presented in Fig. 9.

**Figure 10:**
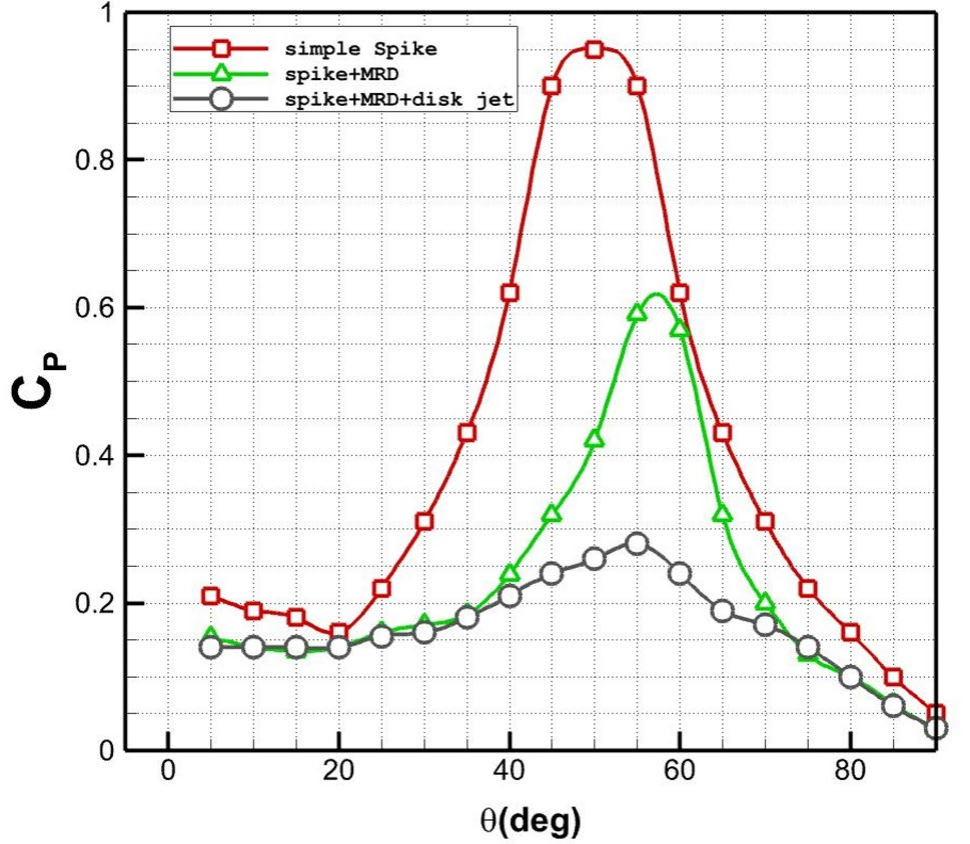
Influence of the different coolant injection systems on Pressure coefficient distribution.

Figure 10 illustrates the variation of the pressure coefficient on the main body for nose cone with/without MRD and disk helium jet. The presence of the spike on the nose cone decreases pressure up to 33% on the main body and the shifts the maximum pressure to higher angles because of the deflection of the air stream. Maximum pressure drops about 50% by injection of the coolant disk jet (C_2_) at angle of 55 deg. Comparison of these models indicates that the helium jet pressure could significantly influence on the deflection of the main shocks.

**Figure 11:**
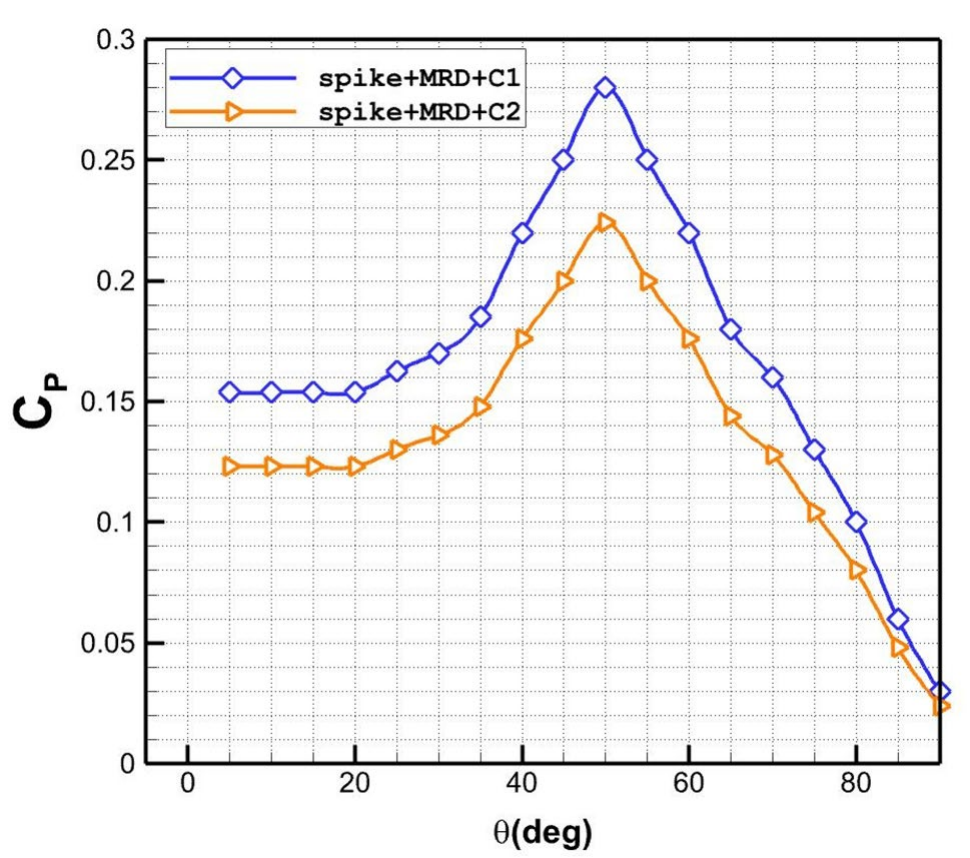
Influence of the different disk coolant injection systems on Pressure coefficient distribution.

The effects of two disk injection configuration on the pressure coefficient distribution on the nose cone are displayed in Fig. 11. Comparison pressure coefficient indicates that injection near the main body is more efficient for the reduction of the pressure on the main body. Since injection from disk 2 (C_2_) is near the main body, it is more helpful for deflection of the main stream and blockage the incoming air. It is found that the influence of shear layer deflection is important on the pressure distribution.

## Conclusion

In the present work, the injection of lateral and disk injection system on the cooling performance of the nose cone with spike at hypersonic flow are extensively investigated. The effects of various coolant gases (helium and carbon dioxide) on the heat load reduction of the main body and spike are fully explained. Computational technique of CFD is used for the modeling of supersonic air flow around the nose cone with multi-row disked spike. The cooling mechanism of these injection systems is disclosed in present work. Three-dimensional model is used for present study to consider real flow physic associated with proposed injection systems. Comparison of the disk injection shows that the coolant jet effectively decreases the temperature nearby the main body while the heat transfer on the spike and disk is not changes. The main advantageous of disk injection is less interaction with main bow shock and high concentration nearby the main body.

## Data Availability

All data generated or analysed during this study are included in this published article.
